# Timing and Risk Factors for a Positive Fecal Immunochemical Test in Subsequent Screening for Colorectal Neoplasms

**DOI:** 10.1371/journal.pone.0136890

**Published:** 2015-09-02

**Authors:** Tsung-Hsien Chiang, Yi-Chia Lee, Wan-Chung Liao, Jui-Hung Chung, Han-Mo Chiu, Chia-Hung Tu, Su-Chiu Chen, Ming-Shiang Wu

**Affiliations:** 1 Department of Internal Medicine, College of Medicine, National Taiwan University, Taipei, Taiwan; 2 Graduate Institute of Clinical Medicine, College of Medicine, National Taiwan University, Taipei, Taiwan; 3 Department of Integrated Diagnostics and Therapeutics, National Taiwan University Hospital, Taipei, Taiwan; 4 Department of Health Care Management, National Taipei University of Nursing and Health Sciences, Taipei, Taiwan; 5 Department of Primary Care Medicine, College of Medicine, National Taiwan University, Taipei, Taiwan; National Cancer Center, JAPAN

## Abstract

**Background:**

Following a negative test, the performance of fecal immunochemical testing in the subsequent screening round is rarely reported. It is crucial to allocate resources to participants who are more likely to test positive subsequently following an initial negative result.

**Objective:**

To identify risk factors associated with a positive result in subsequent screening.

**Methods:**

Dataset was composed of consecutive participants who voluntarily underwent fecal tests and colonoscopy in a routine medical examination at the National Taiwan University Hospital between January 2007 and December 2011. Risk factor assessment of positive fecal test in subsequent screening was performed by using the Cox proportional hazards models.

**Results:**

Our cohort consisted of 3783 participants during a 5-year period. In three rounds of subsequent testing, 3783, 1537, and 624 participants underwent fecal tests, respectively; 5.7%, 5.1%, and 3.9% tested positive, respectively, and the positive predictive values were 40.2%, 20.3%, and 20.8%, respectively. Age ≥60 years (adjusted hazard ratio: 1.53, 95% CI: 1.21–1.93) and male gender (1.32, 95% CI: 1.02–1.69) were risk factors; however, an interaction between age and gender was noted. Men had higher risk than women when they were <60 years of age (*p* = 0.002), while this difference was no longer observed when ≥60 years of age (*p* = 0.74). The optimal interval of screening timing for participant with baseline negative fecal test was 2 years.

**Conclusions:**

Following a negative test, older age and male gender are risk factors for a positive result in the subsequent rounds while the gender difference diminishes with age. Biennial screening is sufficient following a negative fecal test.

## Introduction

Colorectal cancer is the third most common cancer worldwide, and the second leading cause of cancer-related deaths [1]. Studies have shown that population-based screening with either the guaiac-based test or the fecal immunochemical test (FIT) could reduce colorectal cancer mortality by detecting cancers at an earlier stage [2–8]. Compared with the guaiac-based test, the FIT has the advantage of a higher specificity, which may decrease false positive results [9–12].

A major disadvantage of FIT is that the majority of advanced adenomas and early-staged colorectal cancers do not shed a sufficient amount of blood to be detected by the test [9, 13–14]. Theoretically, repeated screening could overcome this issue; however, in the real-life setting, it is common to see the rate of participation decline with time [15]. Furthermore, those in whom advanced adenomas or colorectal cancers were detected might undergo colonoscopic surveillance instead of receiving FIT, so the rate of positive fecal tests also has been found to decline with time [16]. A decrease in both the rate of participation and the rate of positive results would diminish the rate of detection and the overall effectiveness of screening. Therefore, following an initial negative result, it is crucial to identify participants who had a greater risk of a positive result so that the resources of screening can be allocated more effectively.

Presently the test in the majority of the screening programs is the quantitative FIT. However, a qualitative FIT is commercially available and compared with a quantitative FIT [17], a qualitative FIT has the advantage of being cheaper, simpler, and office-based [9]; however, there are few studies to address the performance of such a qualitative test in a screening program, especially with a lower predefined cutoff concentration [18].

Therefore, in this study, we evaluated the test performance of a qualitative FIT in a population-based screening program and sought to identify the risk factors associated with a subsequent positive test by evaluating demographics, education levels, exercise habits, tobacco smoking and alcohol use, co-morbidities, medications, family histories, anthropometric measures, and biochemical studies. We also analyzed the mean interval person-years between positive and negative result of subsequent FIT.

## Methods

### Ethics statement

The study protocol and a letter of consent were approved by the Institutional Review Board of National Taiwan University Hospital (no. 201203096RIC). Written informed consent was obtained from each participant prior to the enrollment.

### Design and participants

Our dataset was composed of consecutive participants who voluntarily underwent FIT and colonoscopy in a routine medical examination at the National Taiwan University Hospital (Health Management Center, Taipei, Taiwan) between January 2007 and December 2011. Participants were recruited through advertisements for health promotion purposes. Before the screening, a self-administered questionnaire was used to collect personal information. On the screening day, participants submitted the FIT and underwent anthropometric measurement, face-to-face interview, blood chemistry tests, and colonoscopy.

Participants with a negative result of FIT in the first screening round were included in the evaluation of risk factors. We excluded those in whom colorectal cancers were detected with the first screening test, those who reported a history of colorectal cancer or inflammatory bowel disease, and those who had an incomplete colonoscopy or poor bowel preparation. According to the recommendation from American College of Gastroenterology [19] and the policy of our nationwide colorectal cancer screening program [20], only participants aged 50 years and more were included in our study.

### Personal profile

We collected the personal profile data, which included demographic data, educational level, anthropometric measures, co-morbid disease (e.g., hypertension, diabetes mellitus, coronary artery disease, and dyslipidemia), medications (e.g., low-dose acetylsalicylic acid, clopidogrel, non-steroidal anti-inflammatory drugs, anti-hypertensive drugs, anti-diabetic drugs, anti-lipid drugs, and uric acid lowering drugs), tobacco smoking and alcohol use, physical exercise habits, family history of cancers (e.g., colorectal, liver, lung, esophagus, breast, nasopharynx, prostate, and stomach), laboratory studies (e.g., hemograms and biochemistries), and baseline colonoscopic findings.

### Fecal immunochemical test

We followed the FITTER checklist [21] for the reporting of our study using fecal immunochemical tests (FIT) for hemoglobin and the details were presented (S1 Checklist). We used a qualitative FIT kit (an immunochromatographic test, OC-Light, Eiken Chemical Co. Ltd., Tokyo, Japan) with a single-day sampling method. The mass of feces collected and volume of the device buffer were claimed as 10mg and 2mL, respectively, for OC-Light. The cutoff concentration for a positive result was claimed at 50 ng hemoglobin (Hb)/mL buffer or more (equivalent to at least 10 micrograms hemoglobin per gram of feces). Participants were advised to collect their fecal samples at home within 2 days before starting bowel preparation for colonoscopy, using the sampler in at least 5 different areas of the feces. Fecal samples were to be placed in collection tube, stored in the refrigerator and submitted to the hospital on the screening day. All fecal samples were tested once and completed at the accredited hospital central laboratory within the screening day.

### Colonoscopy

Colonoscopy was performed by one of nine experienced endoscopists, each with a minimum experience of 5000 colonoscopies. Endoscopic findings were recorded on a standardized computer reporting system. They included information on the quality of bowel preparation, cecal intubation, and location, size, and morphology of the colorectal lesions if present. All the resected specimens were sent for pathological examination. The histopathology of colorectal neoplasia was classified according to the criteria of the World Health Organization [22], which included histologically confirmed colorectal cancer, advanced adenoma (at least 10 mm in diameter, or had high-grade dysplasia, or had villous or tubulovillous histologic characteristics, or any combination thereof), and non-advanced adenoma.

### Statistical analysis

For descriptive findings, the quantitative data are presented as mean ± standard deviation, and categorical variables are presented as percentages. We first evaluated the performance of the fecal immunochemical test using the baseline screening results. We used the fecal test results and colonoscopic findings to construct a 2×2 table, and calculated the sensitivity, specificity, positive and negative predictive values, positive and negative likelihood ratios, accuracy, and the corresponding 95% confidence intervals. Second, after excluding those who tested positive in the baseline screening, we evaluated risk factors associating with a positive result in the subsequent screening. We compared the personal profile of those who tested positive with that of those who tested negative in the subsequent screening using *t*-test or χ^2^ test. We also analyzed the time-to-event data by using the univariate and multivariate Cox proportional hazards models. The results were expressed as hazard ratios and the corresponding 95% confidence intervals. A hazard ratio greater than 1.0 indicated an increased risk for a positive test. Interaction terms were also evaluated in the multivariate model. We evaluated the model discrimination based on the area under the receiver operator curve and the Hosmer-Lemeshow test was applied to assess the calibration performance of the model for goodness of fit.

When significant interaction was found, we performed stratified analyses with the Kaplan-Meier survival curve analysis and the log-rank test. All statistical analyses were conducted using a statistical software package (SAS version 9.2; SAS Institute Inc., Cary, NC). A two-tailed *P* value < 0.05 indicated statistical significance.

With regard to the definition of risk factors, we measured the body mass index as weight in kilograms divided by the square of height in meters, and categorized it in 4 levels. According to the modified Asia criteria [23], metabolic syndrome was defined as the presence of three or more of the following factors: waist circumference >90 cm in men or >80 cm in women, blood triglyceride concentration ≥150 mg/dL, blood high-density lipoprotein cholesterol concentration <40 mg/dL, blood pressure ≥130/85 mmHg or the use of anti-hypertensive drugs, and fasting blood glucose concentration ≥110 mg/dL or blood glycated hemoglobin >6.0%, or the use of anti-diabetic drugs. Anti-platelet agents included low-dose acetylsalicylic acid and clopidogrel. A lower hemoglobin concentration was defined as <12 g/dL in women and <13 g/dL in men. A lower platelet concentration was defined as <150×10^3^/uL. A higher alanine aminotransferase concentration was defined as ≥41U/L. A higher low-density lipoprotein cholesterol concentration was defined as ≥130 mg/dL or the use of anti-lipid drugs. A higher uric acid concentration was defined as >7.5 mg/dL or the use of uric acid lowering drugs. A higher C-reactive protein concentration was defined as ≥0.8 mg/dL. Positive family history of cancer was defined as the presence of specific cancer in third-degree or closer relatives. During the study period, some participants underwent screening colonoscopy with removal of the colonic adenoma; we also included this information in the regression model.

## Results

### Study participants

**Fig 1:**
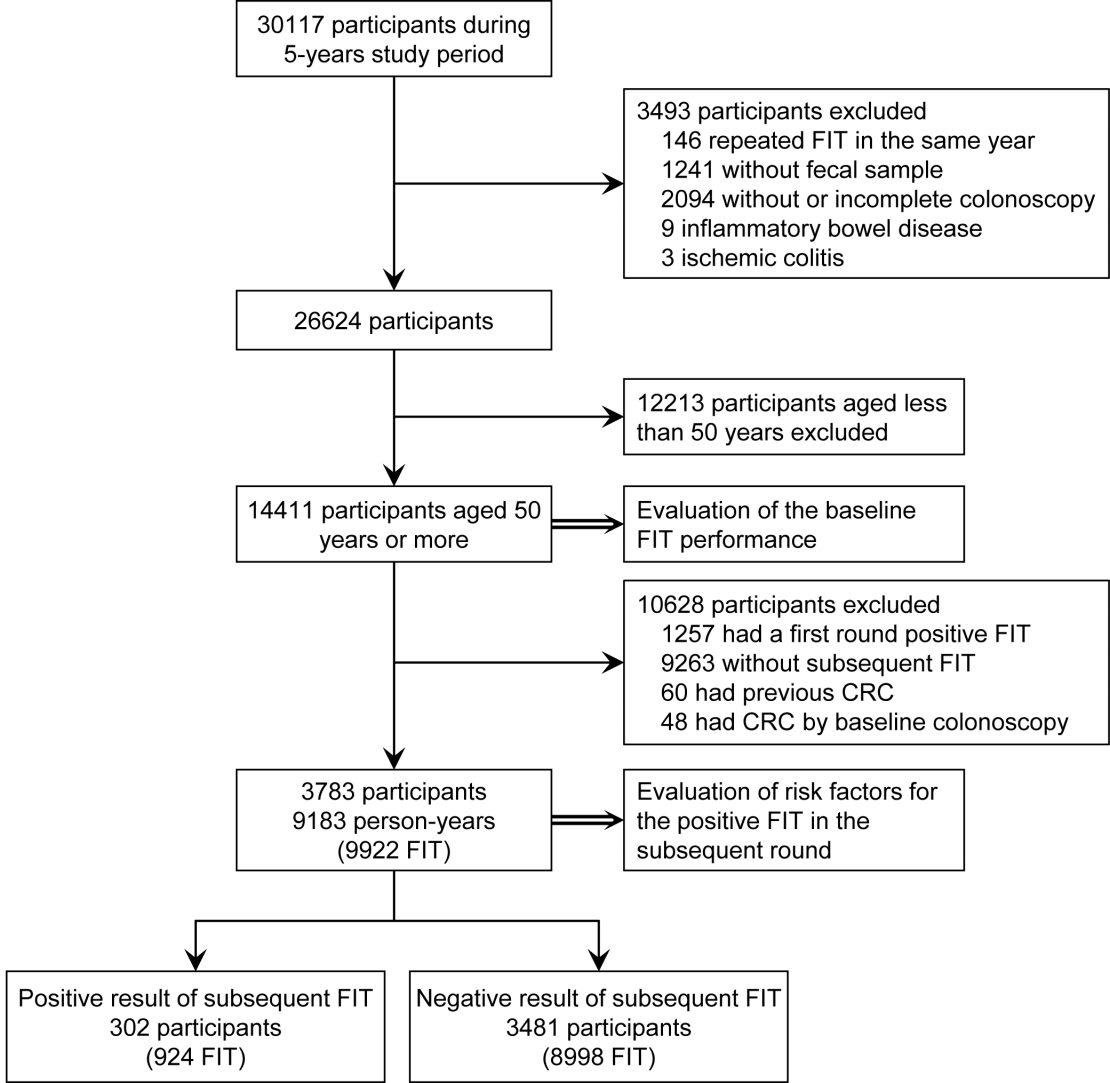
Flow diagram of enrollment.

The study flow is shown in Fig 1. Among 30117 participants with 45625 fecal tests, a total of 14411 participants were included in the evaluation of baseline FIT performance, and 3783 participants who participated in subsequent rounds were included in the risk factor analyses. There were 14411 (54.8% male), 3783 (63.6% male), 1537 (69.9% male), and 624 (74.2% male) participants participating in the first, second, third, and fourth rounds, respectively, and their mean ages were similarly around 59 years. During a 5-year follow-up period with 9922 fecal tests and 9183 person-years, 302 participants tested positive.

### Fecal test performance

The baseline and subsequent performance of FIT are shown in Table 1. From the first to the fourth round, the positive rates of FIT were 8.7%, 5.7%, 5.1%, and 3.9%, respectively, showing a decline with time (*P*<0.001 for trend). For the positive predictive value, the results were 36.1%, 40.2%, 20.3%, and 20.8%, respectively, also showing a decline with time (*P* = 0.034 for trend). The accuracy of all four rounds remained constant.

**Table 1:** Baseline and subsequent performance of fecal immunochemical tests and the corresponding 95% confidence interval in the prediction of colon neoplasms.

Round no.	No.	No. of FIT positivity	Sensitivity	Specificity	PPV	NPV	PLR	NLR	Accuracy
	(male, %)	(%)	(%, 95% CI)	(%, 95% CI)	(%, 95% CI)	(%, 95% CI)	(95% CI)	(95% CI)	(%, 95% CI)
Baseline	14411	1254	14.9	92.9	36.1	80.3	2.11	0.92	76.5
	(54.8)	(8.7)	(13.7–16.2)	(92.5–93.4)	(35.2–36.9)	(79.7–80.9)	(1.90–2.36)	(0.90–0.93)	(75.8–77.2)
2^nd^	3783	214	9.1	95.5	40.2	75.9	2.01	0.95	73.9
	(63.6)	(5.7)	(7.4–11.1)	(94.7–96.2)	(38.6–41.8)	(74.5–77.3)	(1.55–2.62)	(0.93–0.97)	(72.5–75.3)
3^rd^	1537	79	4.5	94.7	20.3	76.5	0.83	1.01	73.6
	(69.9)	(5.1)	(2.8–7.1)	(93.2–95.8)	(18.3–22.3)	(74.4–78.6)	(0.49–1.42)	(0.98–1.04)	(71.4–75.8)
4^th^	624	24	3.5	96.1	20.8	77.2	0.89	1.00	75.0
	(74.2)	(3.9)	(1.5–8.0)	(93.9–97.5)	(17.6–24.0)	(73.9–80.5)	(0.34–2.35)	(0.97–1.04)	(71.6–78.4)

Abbreviation: FIT = fecal immunochemical test; CI = confidence interval; PPV = positive predictive value; NPV = negative predictive value; PLR = positive likelihood ratio; NLR = negative likelihood ratio.

### Risk factors associated with a positive fecal immunochemical test given a first-round negative result

**Fig 2:**
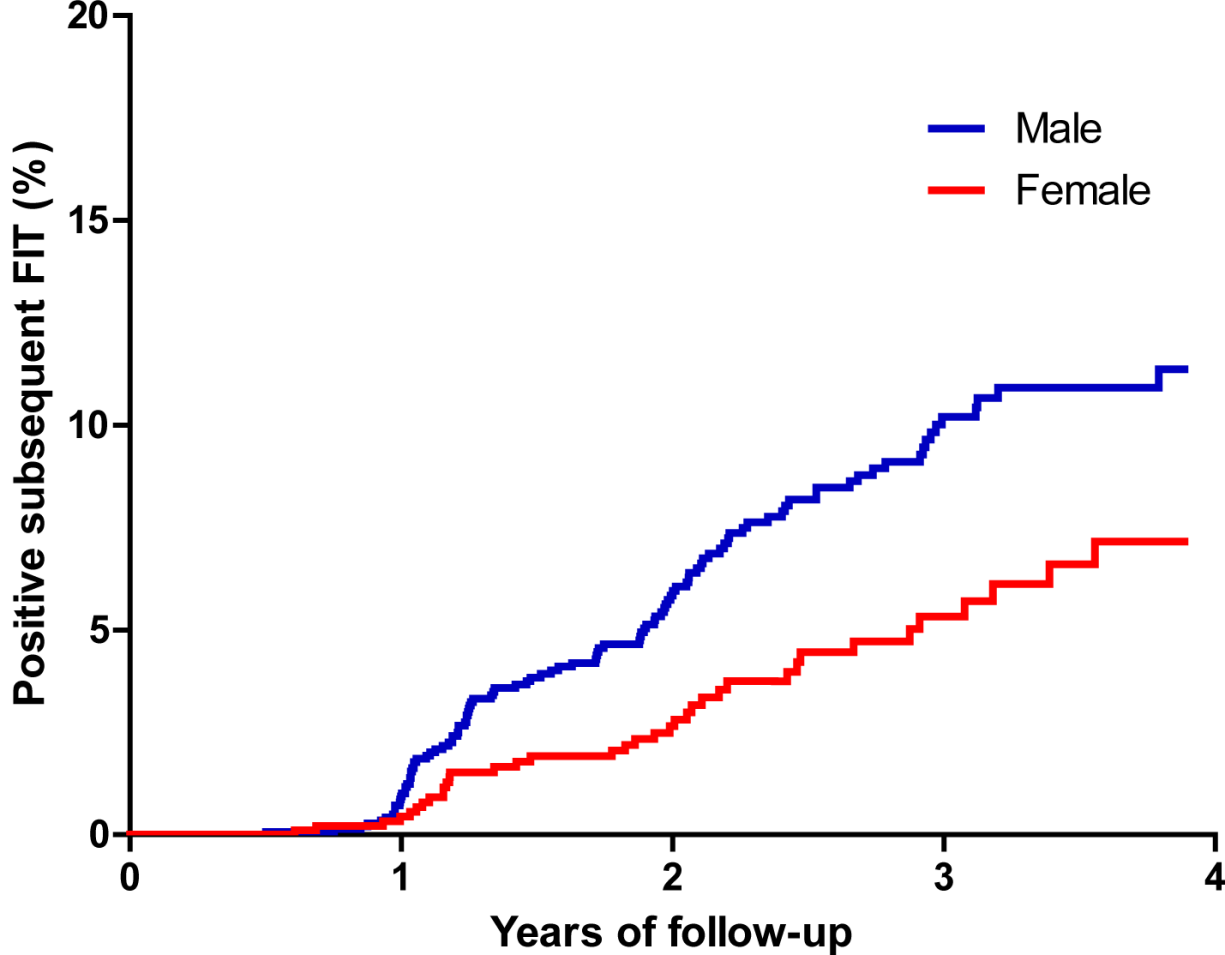
Kaplan-Meier survival analyses for the risk of positive fecal immunochemical tests in the subsequent screening between men and women less than 60 years of age (χ^2^_log-rank_ = 9.74, *P* = 0.002).

**Fig 3:**
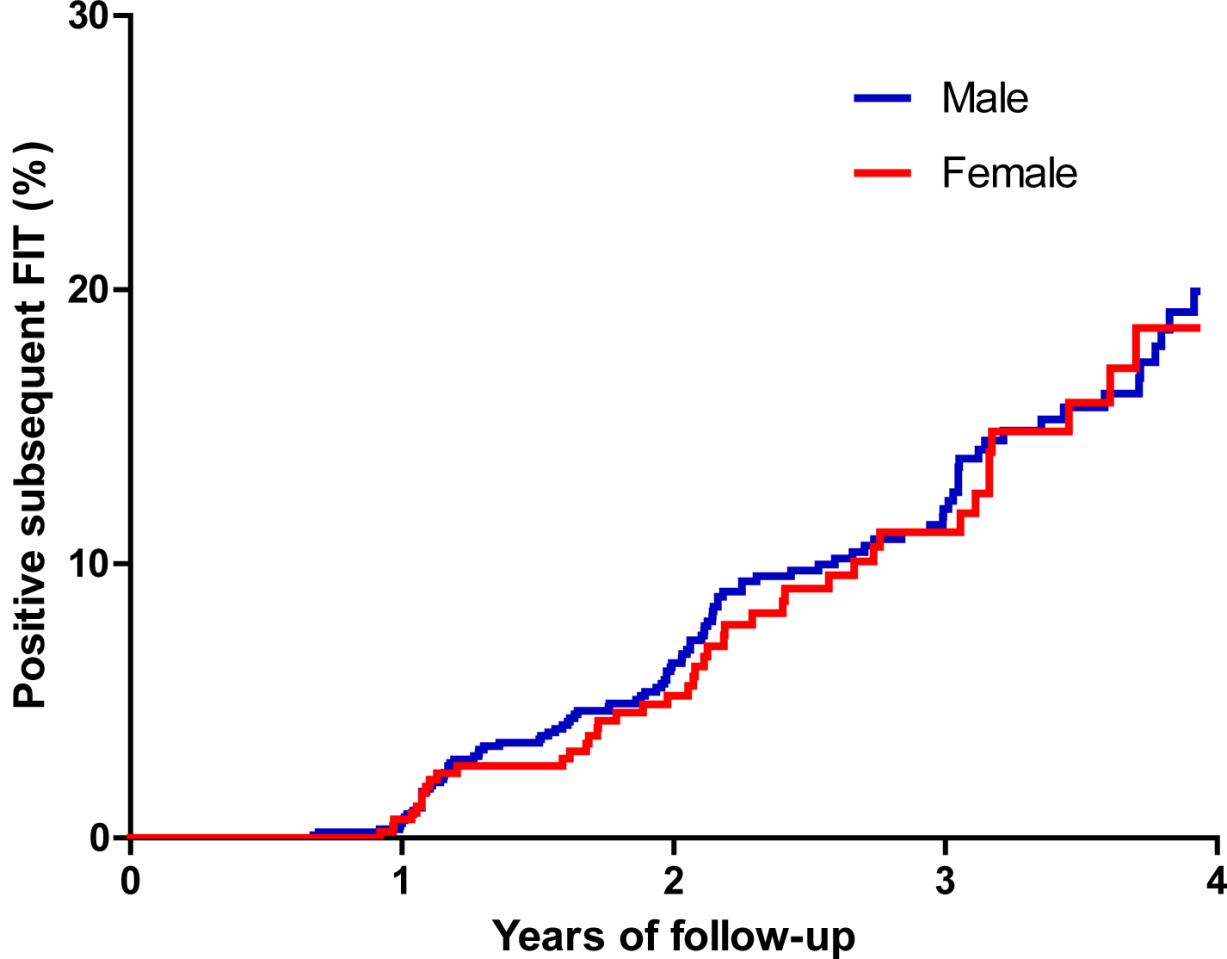
Kaplan-Meier survival analyses for the risk of positive fecal immunochemical tests in the subsequent screening between men and women more than 60 years of age (χ^2^_log-rank_ = 0.11, *P* = 0.74).

Univariate analyses showed that older age, male gender, hypertension, use of anti-platelet agents, higher fasting blood glucose, metabolic syndrome, and detection and resection of colonic adenoma during the study period were significantly associated with a positive result of FIT during the subsequent screening (Table 2). Adjusting for the detection and resection of a colonic adenoma during the study period, the Cox proportional hazards models analyses (Table 3) showed that older age and male gender were significant. The C index of the multivariate model was 0.61 and the model fitting was satisfactory (Hosmer-Lemeshow test, *P* = 0.819). Besides, there was a significant interaction between age and gender (*P* = 0.001). Stratified analyses showed that men <60 years had a greater risk for a positive FIT than did women (*P* = 0.002) (Fig 2) while this difference was no longer observed when participants of both genders were ≥60 years of age (*P* = 0.74) (Fig 3).

**Table 2:** Characteristics of study participants stratified by the results of fecal immunochemical tests in the subsequent rounds.

	Subsequent FIT results			
Characteristics	Positive *n* = 302	Negative *n* = 3481	Total *n* = 3783	*p* value
Age, yr (mean ± SD)	61.4 ± 7.9	59.2 ± 7.1	59.4 ± 7.2	<0.001*
Gender, no. (%)				
Female	87 (28.8)	1290 (37.1)	1377 (36.4)	0.004*
Male	215 (71.2)	2191 (62.9)	2406 (63.6)	
Education level, no. (%)				
≤12 years of schooling	99 (32.8)	1264 (36.3)	1363 (36.0)	0.22
>12 years of schooling	203 (67.2)	2217 (63.7)	2420 (64.0)	
BMI, kg/m^2^, no. (%)				
BMI<18.5	2 (0.7)	68 (2.0)	70 (1.8)	0.40
18.5≤BMI<24	136 (45.0)	1581 (45.4)	1717 (45.4)	
24≤BMI<27	105 (34.8)	1211 (34.8)	1316 (34.8)	
BMI≥27	59 (19.5)	621 (17.8)	680 (18.0)	
Social habits, no. (%)				
Current smoker	35 (11.6)	302 (9.1)	337 (8.9)	0.09
Regular alcohol drinking	38 (12.6)	413 (11.9)	451 (11.9)	0.71
Regular exercise	127 (42.1)	1399 (40.2)	1526 (40.3)	0.53
Co-morbid disease, no. (%)				
Hypertension	97 (32.1)	868 (24.9)	965 (25.5)	0.006*
Diabetes mellitus	30 (9.9)	254 (7.3)	284 (7.5)	0.10
Coronary artery disease	13 (4.3)	125 (3.6)	138 (3.7)	0.53
Dyslipidemia	31 (10.3)	456 (13.1)	487 (12.9)	0.16
Regular use of drugs, no. (%)				
Anti-platelet agents	34 (11.3)	278 (8.0)	312 (8.3)	0.047*
NSAID	14 (4.6)	112 (3.2)	126 (3.3)	0.19
Family history with cancers, no. (%)				
Colorectal cancer	34 (11.3)	451 (13.0)	485 (12.8)	0.40
Liver cancer	38 (12.6)	423 (12.2)	461 (12.2)	0.83
Lung cancer	36 (11.9)	323 (9.3)	359 (9.5)	0.13
Esophageal cancer	8 (2.7)	67 (1.9)	75 (2.0)	0.39
Breast cancer	21 (7.0)	240 (6.9)	261 (6.9)	0.97
Nasopharyngeal cancer	10 (3.3)	110 (3.2)	120 (3.2)	0.89
Prostate cancer	8 (2.7)	100 (2.9)	108 (2.9)	0.82
Gastric cancer	23 (7.6)	290 (8.3)	313 (8.3)	0.67
Abnormal blood tests, no. (%)				
Lower hemoglobin, g/dL	16 (5.3)	211 (6.1)	227 (6.0)	0.59
Lower platelet count, 10^3^/uL	12 (4.0)	142 (4.1)	154 (4.1)	0.93
Higher fasting blood glucose, mg/dL	82 (27.2)	708 (20.3)	790 (20.9)	0.005*
Higher HbA1c, %	59 (19.5)	588 (16.9)	647 (17.1)	0.24
Higher alanine aminotransferase, U/L	46 (15.2)	469 (13.5)	515 (13.6)	0.39
Higher triglyceride, mg/dL	85 (28.2)	827 (23.8)	912 (24.1)	0.09
Lower HDL-C, mg/dL	84 (27.8)	926 (26.6)	1010 (26.7)	0.65
Higher LDL-C, mg/dL	111 (36.8)	1364 (39.2)	1475 (39.0)	0.41
Higher uric acid, mg/dL	53 (17.6)	492 (14.1)	545 (14.4)	0.11
Higher C-reactive protein, mg/dL	2 (0.7)	75 (2.2)	77 (2.0)	0.08
Metabolic syndrome, no. (%)	79 (26.2)	719 (20.7)	798 (21.1)	0.025*
Endoscopic findings				
Baseline with colonic neoplasms, no. (%)	77 (25.5)	723 (20.8)	800 (21.2)	0.05
Colonic adenoma detected and removed during the study period, no. (%)	30 (9.9)	122 (3.5)	152 (4.0)	<0.001*

Abbreviation: SD = standard deviation; BMI = body mass index; FIT = fecal immunochemical test; NSAID = non-steroidal anti-inflammatory drug; HbA1c = glycated hemoglobin; HDL-C = high-density lipoprotein cholesterol; LDL-C = low-density lipoprotein cholesterol.

*p<0.05 indicated statistical significance.

**Table 3:** Cox proportional hazards models to identify risk factors associated with a positive fecal immunochemical test in the subsequent rounds.

			Univariate analyses			Multivariate analyses		
Risk factors		no.	Crude HR	95% CI	*p* value	Adjusted HR	95% CI	*p* value
Age (numerical)	≥50 years	3783	1.04	1.02–1.05	<0.001*			
Age (categorical)	<60 years	2381	1			1		
	60 years	1402	1.66	1.33–2.08	<0.001*	1.53	1.21–1.93	<0.001*
Gender	Female	1377	1			1		
	Male	2406	1.45	1.13–1.86	0.004*	1.32	1.02–1.69	0.034*
Metabolic syndrome	No	2985	1			1		
	Yes	798	1.32	1.02–1.71	0.034*	1.02	0.74–1.41	0.90
Hypertension	No	2818	1			1		
	Yes	965	1.38	1.08–1.76	0.009*	1.14	0.87–1.49	0.35
Anti-platelet agent user	No	3471	1					
	Yes	312	1.40	0.98–2.01	0.06			
Abnormal fasting blood glucose	No	2993	1			1		
	Yes	790	1.48	1.15–1.91	0.003*	1.26	0.93–1.69	0.13
Colonic adenoma detected and removed during the study period	No	3631	1			1		
	Yes	152	1.94	1.33–2.83	<0.001*	1.76	1.20–2.57	0.004*

Abbreviation: HR = hazard ratio; CI = confidence interval.

Note that only the categorical age was entered into the multivariate Cox proportional hazards models.

*p<0.05 indicated statistical significance.

### Optimal timing of subsequent fecal immunochemical test

The mean number of screening rounds was 2.62 ± 0.88, and the mean interval between successive rounds of screening was 2.43 ± 1.01 years. Regardless of age and gender, the mean interval person-years for positive result of subsequent FIT were around 2 years and significant shorter than negative result (Table 4).

**Table 4:** Person-years stratify with age and gender by subsequent fecal immunochemical test results.

	Person-years at risk	Person-years by FIT results (mean ± SD)		
		Positive	Negative	*p* value
Male				
<60	3490	1.83 ± 0.85	2.44 ± 1.04	<0.001*
≧60	2328	2.19 ± 1.00	2.48 ± 1.00	0.005*
Subtotal	5818	2.00 ± 0.94	2.45 ± 1.02	<0.001*
Female				
<60	2267	2.08 ± 1.02	2.46 ± 0.99	0.014*
≧60	1098	2.26 ± 1.03	2.46 ± 0.97	0.20
Subtotal	3365	2.17 ± 1.02	2.46 ± 0.98	0.009*
Both genders				
<60	5757	1.90 ± 0.90	2.45 ± 1.02	<0.001*
≧60	3426	2.21 ± 1.00	2.47 ± 0.99	0.002*
Total	9183	2.06 ± 0.97	2.46 ± 1.01	<0.001*

Abbreviation: FIT = fecal immunochemical test; SD = standard deviation.

*p<0.05 indicated statistical significance.

## Discussion

In this study, we collected unique data with a comprehensive profile of individual risk factors to evaluate which factors were associated with a positive result in subsequent rounds of FIT. Following a negative result in the first round, we found that older age and male gender were associated with a positive test result in a subsequent round; however, in participants over 60 years of age, the gender difference was no longer seen. We also found an inter-screening interval of 2 years may be sufficient following a negative fecal test. Together, our findings suggest that timing and selective invitation according to age and gender may be a useful approach for better resource allocation in subsequent screening.

In our study (*n* = 3783 at baseline) based on the qualitative FIT with the cutoff concentration of 50 ng Hb/mL buffer, we found that both the positive rates and positive predictive values of FIT declined with time. During the subsequent four rounds of screening, our positive rates of FIT were 8.7%, 5.7%, 5.1%, and 3.9%, respectively, and the positive predictive values for colorectal neoplasia were 36.1%, 40.2%, 20.3%, and 20.8%, respectively. Our findings were consistent with those of the previous study conducted in Netherlands (*n* = 2871 at baseline) [24] using the quantitative FIT with the cutoff concentration of 50 ng Hb/mL buffer. They showed that the positive rate of FIT for the first and second round were 8.1% and 7.4%, respectively. For participants who tested negative in the first round and participated in the second round, the positive predictive values declined from 55% to 44% for advanced neoplasia, and from 8% to 4% for colorectal cancer. However, another study (*n* = 2959 at baseline) conducted in Italy using the quantitative FIT with the cutoff concentration of 100 ng Hb/mL buffer did not show such a decline [16]. Instead, they found that during the subsequent four rounds of screening, the participation rates in the four rounds were 56.1%, 62.4%, 57.3%, and 62.6%, respectively, the positive rates of fecal immunochemical tests were 4.3%, 4.2%, 3.7%, and 4.4%, respectively, and the positive predictive values for advanced adenoma were 34.5%, 31.5%, 27.6%, and 33.3%, respectively, a non-significant change. Based on the quantitative FIT using the cutoff concentration of 100 ng Hb/mL buffer, the previous study in Taiwan showed that the positive rate of FIT for the first and subsequent round were 4.0% and 3.8%, respectively [15], and overall positive predictive values for advanced adenoma and colorectal cancer were 13.2% and 6.8%, respectively [17]. Such a discrepancy is keeping with the lower cut-off concentration of the qualitative FIT used in the present study. Clarification would also require further studies with a larger sample size and a longer follow-up period to confirm the durability of repeated screening.

Although current colorectal cancer screening guidelines are not age or gender specific [25], older age and male gender are well known to be associated with a higher risk for colorectal neoplasms [26]. A study conducted in Germany showed that the transition rates from adenoma to colorectal cancer for the age group 55 to 59 years and the group ≥80 years were 2.6% and 5.6%, respectively, for women, and were 2.6% and 5.1%, respectively, for men, indicating a strong age gradient [27]. In another study controlling for sex, age, and birth cohort effect, men were found to reach equivalent prevalence rates of colorectal cancer and advanced neoplasms 3.4 and 6.9 years earlier than women, respectively [28]. A study conducted in Austria found that the prevalence and number needed to screen of advanced adenomas were comparable between men 45 to 49 years of age and women 55 to 59 years of age [29]. Similar findings were seen in a study conducted in the USA, in which, when women were compared with men. In this study, there was a 10-year lag in the prevalence of colon polyps >9 mm [30].

Our study also showed that men showed a greater risk for a positive FIT in subsequent screening; however, we found that this difference occurred mainly when they were <60 years of age. We speculate that the underlying mechanism may be related to the role of estrogen in colorectal tumorigenesis [31]. Colorectal cancer rates have been found reduced by 37% from a clinical trial of using estrogen plus progestin [32]; however, another clinical trial found that women with hormone replacement therapy were diagnosed with more advanced stages of colorectal cancer than were those who received placebo [33]. Further studies are needed to resolve this controversy.

In addition to age and gender, other possible risk factors for colorectal cancer include exercise habit, tobacco smoking and alcohol use, co-morbid diseases, family history, and biochemical markers. It has been suggested that the effectiveness of a cancer-screening program may be improved if the program can be tailored to the individual cancer risk based on the individual risk factors. One meta-analysis suggested a strategy for colorectal cancer screening that would involve lowering the age at initial screening in smokers [34]. Adherence to the recommendations for physical activity, waist circumference, smoking, alcohol intake, and diet has been shown to reduce colorectal cancer risk in a Danish study [35]. A Taiwanese study found a significant impact of metabolic syndrome, smoking and male gender on the risk of colorectal neoplasms [36]. Consistently, in our study in the univariate analysis, we found that age, gender, hypertension, higher fasting blood glucose, the presence of metabolic syndrome, use of anti-platelet agents, and detection and resection of colonic adenoma in the colonoscopic screening were risk factors associated with subsequent positive results with the FIT. In multivariate analysis, age and gender retained their significance. We further demonstrated the sufficient inter-screening interval following a negative fecal test in this subsequent screening program with a lower predefined qualitative FIT. This strategy might be readily applicable in the setting of mass screening program.

The strengths of the current study include the comprehensive nature of the collection of personal profile information, enabling us to evaluate the risk factors during a sufficiently long follow-up period; however, our study has some limitations. First, only about 26% of participants underwent the subsequent screening with the fecal immunochemical tests, so we could not rule out the possibility of self-selection bias in our study without a randomized allocation. Second, the risk factors identified in the present study may be time-dependent so the measurement at baseline may not accurately reflect the impact on the entire follow-up period. Third, although we have extensively evaluated the risk factors for a positive FIT in the subsequent round based on the conventional risk factors, including the demographics, metabolic factors, medications, and the colonoscopic findings in the first screening, the discrimination performance of our model remained unsatisfactory, which may require the input of novel molecular tests to improve the ability in prediction. Finally, since the study was based on a close cohort, we did not consider the dynamic nature that may exist in the community-based screening program, which may limit our generalizability.

In summary, in a population-based screening program with the periodic FIT, our study showed that older age and male gender were risk factors for participants being more likely to have a positive result in the subsequent rounds, and an inter-screening interval of 2 years is sufficient following a negative fecal test. Besides, the interaction between gender and age at the time of screening may have important implications for the design of a tailored screening program according to the individual risk of colorectal neoplasms.

## Supporting Information

S1 FITTER Checklist The FITTER checklist for the reporting of studies using fecal immunochemical tests for hemoglobin. (DOC)
