# Supplementary material for: Timing and Risk Factors for a Positive Fecal Immunochemical Test in Subsequent Screening for Colorectal Neoplasms
Source: PLoS One. 2015 Sep 2;10(9):e0136890. doi: 10.1371/journal.pone.0136890 (PMC4558044; doi:10.1371/journal.pone.0136890)
Supplement: S1 FITTER Checklist — (DOC) [file pone.0136890.s001.doc]

**Supporting Information**

**S1 Checklist.** The FITTER checklist for the reporting of studies using fecal immunochemical tests for hemoglobin

| **Topic** | **Item** | **Priority** | **Documentation** |
| --- | --- | --- | --- |
| **Specimen collection and handling** | | | |
|  | Name of specimen collection device and supplier (address). | Essential | page 9, line 10-11 |
|  | Description of specimen collection device (vial with probe/stick, card, other). | Essential | page 9, line 15-19 |
|  | Description of specimens used if an *in vivo* study (single or pooled feces, artificial matrix with added blood, etc). | Essential for laboratory evaluations | NA |
|  | Details of fecal collection method (sampling technique and number of samples). | Essential | page 9, line 10-11 and 15-19 |
|  | Who collected the specimens from the samples (patient, technician, etc). | Essential | page 9, line 15-17 |
|  | Number of fecal specimens used in the study (single, pooled, individual patient feces). | Essential for laboratory evaluations | page 13, line 7-8 |
|  | Mean mass of feces collected.* | Essential | NA |
|  | Volume of buffer into which specimen is taken by probe, applicator stick or card.* | Essential | page 9, line 11-13 |
|  | Time and storage conditions of fecal specimen from “passing” to sampling, including time and temperature (median and range). | Essential for laboratory evaluations | NA |
|  | Time and storage of collection devices from specimen collection to analysis, including time and temperature (median and range). A concise description of process from collection to analysis is recommended. | Essential | NA |
| **Analysis** | | | |
|  | Name of analyser, model, supplier (address), number of systems if more than one used. | Essential | NA |
|  | Number of times each sample was analysed. | Essential | page 9, line 19 to page 10, line 1 |
|  | Analytical working range* and whether samples outside this range were diluted (factor) and reassayed. | Essential for laboratory evaluations | page 9, line 13-15 |
|  | Source of calibrator(s) (supplier with address), number of calibrator(s), how concentrations were assigned* and details of calibration process including frequency. | Essential for laboratory evaluations | NA |
|  | Analytical imprecision*, ideally with number of samples analysed, concentrations, and mean, SD and CV. | Essential for all studies | NA |
| **Quality management** | | | |
|  | Source (address) or description of internal quality control materials, number of controls, assigned target concentrations and ranges, how target concentrations were assigned, rules used for acceptance and rejection of analytical runs. | Desirable for laboratory evaluations | NA |
|  | Participation in external quality assessment schemes: (name and address of scheme), frequency of challenges, performance attained. | Desirable for laboratory evaluations | NA |
|  | Accreditation held by the analytical facility (address). | Desirable for laboratory evaluations | page 9, line 19 to page 10, line 1 |
|  | The number, training and expertise of the persons performing the analyses and recording the results. | Essential | NA |
| **Result handling** | | | |
|  | Mode of collection of data – manual recording or via automatic download to IT system, single or double reading. | Desirable | NA |
|  | Units used, with conversion to µg Hb/g feces if ng Hb/mL used. | Essential | page 9, line 13-15 |
|  | Cut-off concentration(s) if used and explanation of how assigned locally or by manufacturer.^＊^ | Essential | page 9, line 13-15 |
|  | Were the analysts blinded (masked) to the results of the reference investigation and other clinical information? | Essential | NA |
| **^＊^information available from manufacturer or supplier** | | | |

Note: NA=not applicable
